# Neurogenic Effects of Ghrelin on the Hippocampus

**DOI:** 10.3390/ijms18030588

**Published:** 2017-03-08

**Authors:** Chanyang Kim, Sehee Kim, Seungjoon Park

**Affiliations:** 1 Department of Biomedical Science, Graduate School, Kyung Hee University, Seoul 02447, Korea; praise1107@naver.com (C.K.); shysl@hanmail.net (S.K.); 2 Department of Pharmacology and Medical Research Center for Bioreaction to Reactive Oxygen Species and Biomedical Science Institute, School of Medicine, Kyung Hee University, Seoul 02447, Korea

**Keywords:** ghrelin, hippocampus, neurogenesis, learning, memory

## Abstract

Mammalian neurogenesis continues throughout adulthood in the subventricular zone of the lateral ventricle and in the subgranular zone of the dentate gyrus in the hippocampus. It is well known that hippocampal neurogenesis is essential in mediating hippocampus-dependent learning and memory. Ghrelin, a peptide hormone mainly synthesized in the stomach, has been shown to play a major role in the regulation of energy metabolism. A plethora of evidence indicates that ghrelin can also exert important effects on neurogenesis in the hippocampus of the adult brain. The aim of this review is to discuss the current role of ghrelin on the in vivo and in vitro regulation of neurogenesis in the adult hippocampus. We will also discuss the possible role of ghrelin in dietary restriction-induced hippocampal neurogenesis and the link between ghrelin-induced hippocampal neurogenesis and cognitive functions.

## 1. Introduction

Ghrelin is a unique 28-amino acid peptide hormone mainly produced in the stomach. The acylated form of ghrelin, which is esterified with octanoic acid on Ser3, exerts its effects through the activation of the growth hormone (GH) secretagogue receptor 1a (GHS-R1a) to release GH [1]. Earlier studies found that ghrelin stimulates GH release primarily at the levels of the hypothalamus and anterior pituitary, and increases food intake to regulate energy homeostasis [2]. Ghrelin also has various physiological actions throughout the body, including effects on exocrine and endocrine pancreatic functions, carbohydrate metabolism, the cardiovascular system, gastric secretion, stomach motility, and sleep [3,4,5].

Previous immunocytochemical studies reported that ghrelin-containing neurons were detected in the arcuate nucleus of the hypothalamus [6] and in the cortex [7]. However, current evidence does not support this idea because ghrelin-specific staining was not found in the brain of the rat and mouse [8]. Ghrelin receptor mRNAs are widely detected in different regions of the brain, including the arcuate and ventromedial nuclei of the hypothalamus, cornu ammonis regions 2 and 3 of the hippocampus, and the dentate gyrus (DG) of the hippocampal formation [8,9,10]. It has been shown that peripherally administered ghrelin can pass through the blood-brain barrier, and binding sites for ghrelin are found in the hypothalamus and hippocampus [11]. In the central nervous system, ghrelin is found to have a variety of non-endocrine effects, such as enhancement of cognitive function, attenuation of anxiety and depression, modulation of reward and motivation, and protection of neuronal cells, to control neuronal functions and subsequently alter many brain functions [12].

Neurogenesis is known to continue throughout adulthood in a few mammalian species in the subgranular zone (SGZ) of the DG and in the subventricular zone of the lateral ventricle [13,14]. The hippocampus plays an essential role in the process of learning, memory, and emotional responses [14]. For that reason, a lot of attention has been paid on hippocampal neurogenesis. Indeed, memory deterioration in ischemic injury is associated with neuronal deficit in the hippocampus [15]. Some growth factors, including fibroblast growth factor 2, insulin-like growth factor (IGF)-1, and vascular endothelial growth factor, promote cognitive function through enhancement of generation or survival of new neurons in the hippocampus [16]. Exact understanding of the mechanisms involved in the adult hippocampal neurogenesis may boost the development of new therapy using stem cell-based strategies against neurodegenerative diseases. In this review, we will focus on the actions of acyl-ghrelin on adult neurogenesis in the hippocampus. The role of acyl-ghrelin-induced hippocampal neurogenesis in learning and memory will also be discussed.

## 2. Effects of Ghrelin on Adult Hippocampal Neurogenesis

Several lines of evidence have indicated that ghrelin regulates adult hippocampal neurogenesis in vivo. Earlier studies indicate that ghrelin acts directly on the dorsal motor nucleus of vagus (DMNV) [17] and the nucleus of the solitary tract (NTS) [18] to stimulate neurogenesis in adult rats with cervical vagotomy. Ghrelin also increases the percentage of BrdU incorporation into cultured DMNV and NTS neurons. In addition, des-acyl ghrelin, as well as ghrelin, induces proliferation of neuronal precursor cells that is both dependent and independent of GHS-R1a in the rat fetal spinal cord [19].

In adult male mice peripherally treated with ghrelin, BrdU incorporation and doublecortin (DCX)-positive neuroblasts were increased in the dentate SGZ [20,21]. Moreover, it was found that even endogenous ghrelin plays an important role in neurogenesis because immunoneutralization of ghrelin inhibited BrdU incorporation and decreased DCX-positive cells in the SGZ [20]. Adult hippocampal neurogenesis was also increased in rats [22] and Alzheimer’s disease model 5XFAD mice [23] treated with ghrelin.

Further study using ghrelin knockout mice revealed that targeted disruption of the ghrelin gene results in decreased numbers of progenitor cells in the DG of the hippocampus, while ghrelin replacement restored progenitor cell numbers to those of wild-type controls [24]. In this study, it was also found that not only the number of BrdU-positive cells but also the fraction of immature neurons and newly generated neurons were decreased in the ghrelin knockout mice, which were increased by ghrelin replacement [24]. In addition, a study using rats lacking the ghrelin receptor indicated that impairment in ghrelin signaling exhibits an early onset decrement in hippocampal spine density and neurogenesis [25]. These results suggest the direct involvement of ghrelin in the proliferation and differentiation of adult neural progenitor cells in the SGZ.

However, it should be noted that there is a possibility of indirect effects of ghrelin on hippocampal neurogenesis. Ghrelin effectively stimulates the secretion of GH from the anterior pituitary gland, and subsequently increases hepatic IGF-1 secretion [1]. Peripheral infusions of both GH and IGF-1 induce hippocampal neurogenesis in the adult rat [26,27]. To investigate this possibility, the effect of ghrelin on proliferation and differentiation of progenitor cells in the SGZ of spontaneous dwarf rats, a model for pituitary dwarfism with a point mutation of the GH gene resulting in total loss of GH and decreased IGF-1 levels [28,29], was assessed [30]. In this study, it was found that Ki-67-positive progenitor cells and DCX-positive neuroblasts in the DG of the spontaneous dwarf rats expressed GHS-R1a. In the spontaneous dwarf rats, ghrelin treatment increased the number of proliferating cell nuclear antigen-, BrdU-, and DCX-labeled cells in the DG, suggesting ghrelin regulates adult hippocampal neurogenesis independently of the somatotropic axis.

## 3. Role of Ghrelin in Dietary Restriction-Induced Hippocampal Neurogenesis

Dietary restriction (DR), which gives several beneficial effects [31], is known to increase the survival of new adult born hippocampal cells [32,33,34] and to enhance synaptic plasticity [35]. Plasma ghrelin levels are elevated during DR [36,37,38]. These findings suggest that ghrelin may be a potential mediator of enhancement of hippocampal neurogenesis induced by DR. In order to examine if the augmentation of hippocampal neurogenesis in response to DR is mediated by ghrelin, mice lacking ghrelin were used [39]. In this study, the authors provide evidence that ghrelin is involved in DR-induced hippocampal neurogenesis in adult mice. Specifically, protein levels of gastric and hippocampal ghrelin in wild-type mice were increased after three months of DR. It has been shown that DR promotes the hippocampal neurogenesis by increasing the survival of newborn cells in wild-type mice, as reported previously [32,33,34]. By contrast, in ghrelin-deleted mice, DR did not alter the survival of newly generated cells in the DG. In addition, the beneficial effects of DR on enhancing adult hippocampal neurogenesis were not observed in GHS-R1a knockout mice [40]. These results suggest that elevated levels of ghrelin during DR may have an important role in the enhancement of neurogenesis induced by DR.

## 4. Role of Ghrelin-Induced Hippocampal Neurogenesis in Learning and Memory

Ghrelin is known to play a crucial role in learning and memory processes. Specifically, intracerebroventricular or intrahippocampal injection of ghrelin increases memory retention in rodents [41,42]. Direct administration of ghrelin into the dorsal raphe nucleus also increased memory retention through serotonergic inputs from the dorsal raphe nucleus [43]. Furthermore, treatment of rats with nonpeptide ghrelin agonists induces improvement in the performance of the modified water maze and novel object recognition (NOR) tests [44]. In contrast, it has been reported that central injection of a GHS-R1a-selective antagonist into the rat impairs memory encoding on both acquisition and consolidation stages [45].

Considering that adult hippocampal neurogenesis is correlated with spatial learning and memory process, decreased neurogenesis in ghrelin knockout mice may attribute to impaired cognitive function. Indeed, targeted deletion of ghrelin led to impaired memory performance of mice in the NOR test [11,24]. The ghrelin knockout mice also showed decreased spontaneous alternation behavior in the *Y*-maze task [24], suggesting impairment in their spatial working memory. However, these functional impairments observed in ghrelin knockout mice were rapidly reversed by ghrelin treatment. Moreover, it has been shown that increased adult hippocampal neurogenesis is necessary for improving hippocampus-dependent pattern separation function [46,47]. A recent study showed that long-term daily injections with ghrelin, at a dose similar to physiologic range after a 24 h fast, promoted pattern separation memory performance on the spontaneous location recognition task [22]. These results indicate that ghrelin exerts influences on brain structures responsible for learning and memory performance. In addition, in the spontaneous dwarf rats, ghrelin treatment increased spontaneous alternation rates in the *Y*-maze task and time spent exploring the novel objects in the NOR test compared to vehicle-treated animals [30], suggesting that ghrelin-induced cognitive function enhancement is mediated independently of the GH/IGF-1 axis. Thus, it seems likely that ghrelin-induced hippocampal neurogenesis may play a crucial role in augmenting cognitive functions. In addition, the ability of ghrelin in enhancing learning and memory may be associated with the promotion of hippocampal synaptic plasticity, which is important in memory acquisition [48], by augmenting dendritic spine formation and long-term potentiation [11]. In addition, ghrelin-induced neuroprotection in the hippocampus [49,50] appears to contribute to its enhancing effects on learning and memory.

It also has been reported that enhanced performance on a hippocampal-dependent learning and memory task [51,52] and increased numbers of newly formed cells in the DG due to enhanced cell survival [33] are observed in calorie restricted adult animals, where circulating plasma ghrelin levels are increased [36,37,38].

## 5. Multiple Pathways Are Involved in Ghrelin-Induced Proliferation of Hippocampal Neural Stem Cells

An earlier study reported that ghrelin increases cellular proliferation of adult rat hippocampal progenitor cells in vitro [53]. The proliferative effect of ghrelin in adult rat hippocampal neural stem cells (NSCs) appears to be mediated through the activation of GHS-R1a because a receptor-specific antagonist completely blocked the effect of ghrelin [54]. Ghrelin increased the activation of extracellular signal-regulated kinase (ERK) 1/2 and Akt [54], which play important roles in regulating the proliferation of neural progenitor cells [55,56,57]. Treatment of hippocampal NSCs to the inhibitors of MEK/ERK1/2 and phosphatidylinositol-3-kinase (PI3K)/Akt resulted in suppression of the ghrelin-induced proliferative effect [54]. These results suggest that ghrelin increased proliferation of hippocampal NSCs through the activation of MEK/ERK1/2 and PI3K/Akt pathways. Ghrelin also enhanced phosphorylation of Akt downstream effectors, such as glycogen synthase kinase (GSK)-3β, mammalian target of rapamycin (mTOR), and p70^S6K^. In addition, ghrelin treatment led to an activation of signal transducer and activator of transcription (STAT) 3. Moreover, pretreatment of hippocampal NSCs with specific inhibitors of mTOR and Jak2/STAT3 attenuated ghrelin-induced cell proliferation [54]. Taken together, these findings suggest that numerous signaling pathways, such as MEK/ERK1/2, PI3K/Akt/GSK-3β, PI3K/Akt/mTOR/p70^S6K^, and Jak2/STAT3, are involved in ghrelin-induced proliferation of hippocampal NSCs. It is still unclear whether these same signaling pathways operate in vivo.

Additionally, in a recent paper [58], it was shown that ghrelin-induced mitogenic regulation is mediated via enhanced progression from G_1_ to S phase due to the augmented expression of transcription factor E2F1 in the nucleus. The ghrelin-induced proliferative effect appears to be mediated by increased expression of genes that promote the cell cycle, such as cyclin A and cyclin-dependent kinase (CDK) 2 [58]. Moreover, ghrelin treatment induced a decrease in protein levels of cell cycle negative regulators, such as p27^KIP1^ and p57^KIP2^, suggesting downregulation of CDK inhibitors may contribute to the proliferative effect of ghrelin. Ghrelin-mediated cell cycle progression from G_0_/G_1_ to S phase was dependent on the activities of MEK/ERK1/2, PI3K/Akt/mTOR, and JAK2/STAT3 signaling pathways [58].

## 6. Conclusions

This review clearly shows that ghrelin is involved in the induction of the proliferation, survival, and differentiation of adult hippocampal neural progenitor cells (Table 1). Ghrelin-induced adult neurogenesis in the hippocampus is mediated independently of the GH/IGF-1 axis. In vitro studies indicate that multiple signaling pathways are involved in the mediation of the actions of ghrelin on hippocampal neurogenesis. The effects of ghrelin on hippocampal neurogenesis combined with its stimulatory actions on synaptic plasticity may help to improve hippocampus-dependent cognitive function. Future studies will be needed to translate data gained from animal and in vitro experiments into clinical applications.

## Figures and Tables

**Table 1:** Summary of the effects of ghrelin on adult hippocampal neurogenesis.

Experimental Models	Key Findings	References
C57Bl/6 mice	Ghrelin increased BrdU+ and DCX+ cells. Anti-ghrelin antibody decreased BrdU+ and DCX+ cells.	Moon et al., 2009 [20]
Ghrelin knockout mice	Targeted deletion of ghrelin gene decreased PCNA+, Ki-67+, BrdU+, BrdU+/DCX+, and BrdU+/NeuN+ cells, which were increased by ghrelin replacement.	Li et al., 2013 [24]
Spontaneous dwarf rats	Ghrelin increased PCNA+, BrdU+, and DCX+ cells.	Li et al., 2013 [30]
5XFAD (Alzheimer’s disease model) mice	Ghrelin increased BrdU+, HH3+, and calretinin+ cells.	Moon et al., 2014 [23]
C57Bl/6 mice	Ghrelin increased BrdU+, BrdU+/DCX+, and BrdU+/NeuN+ cells.	Zhao et al., 2014 [21]
Ghrelin knockout mice	Dietary restriction increased survival of newly generated cells in wild-type mice, but not in ghrelin knockout mice.	Kim et al., 2015 [39]
Lister Hooded rats	Ghrelin increased BrdU+, DCX+, and BrdU+/NeuN+ cells.	Kent et al., 2015 [22]
Adult rat hippocampal neural stem cells	Ghrelin increased ^3^[H] incorporation.	Johansson et al., 2008 [53]
Adult rat hippocampal neural stem cells	Ghrelin increased the proliferation through the activation of GHS-R1a. MEK/ERK1/2, PI3K/Akt/GSK-3β, PI3K/Akt/mTOR/p70^S6K^, and Jaks/STAT3 signaling pathways were involved in ghrelin-induced proliferative effects.	Chung et al., 2013 [54]
Adult rat hippocampal neural stem cells	Ghrelin increased expression of genes that promote the cell cycle, while inhibiting expression of negative regulators of the cell cycle.	Chung and Park, 2016 [58]
